# Unique Clinical and Psychiatric Challenges in Elderly Patients With Guillain-Barré Syndrome: A Case Series

**DOI:** 10.7759/cureus.69478

**Published:** 2024-09-15

**Authors:** Koji Obara

**Affiliations:** 1 Neurology, National Hospital Organization Akita National Hospital, Yurihonjo, JPN; 2 Neurology, Yasumi Hospital, Morioka, JPN

**Keywords:** akinesia, axonal neuropathy, elderly, guillain-barré syndrome, psychiatric symptoms

## Abstract

Guillain-Barré syndrome (GBS) is an immune-mediated peripheral neuropathy characterized by rapid-onset bilateral flaccid paralysis and areflexia, often triggered by an antecedent illness. Here, we report three elderly male patients, aged 67, 78, and 81, who developed severe GBS requiring mechanical ventilation. Each patient presented with rapid neurological deterioration following antecedent illnesses, such as respiratory and gastrointestinal infections. Cranial nerve involvement, particularly ocular movement deficits, was prominent. Nerve conduction studies revealed motor and sensory axonopathy and cerebrospinal fluid analysis showed albuminocytologic dissociation. Despite initial treatment with intravenous immunoglobulin and plasmapheresis, all patients developed akinesia in the chronic phase. One patient was diagnosed with progressive supranuclear palsy, while MRI in the other two patients revealed cerebral ischemic lesions and temporal lobe atrophy. Elderly GBS patients tend to have rapid progression, severe neurological deficits, and prolonged recovery. In addition, the disease severity and protracted recovery can lead to stress reactions and consequent psychiatric symptoms in GBS patients. Long-term management strategies, both neurological and psychiatric, are essential for elderly GBS patients.

## Introduction

Guillain-Barré syndrome (GBS) is an immune-mediated peripheral neuropathy characterized by rapid-onset bilateral flaccid paralysis and areflexia, often precipitated by an antecedent illness [1]. While GBS can affect individuals of all ages, recent epidemiological studies suggest a geographically widespread increase in GBS incidence among older adults [2,3]. Elderly patients with GBS tend to have more significant disease severity, a longer time to recover, and a predominance of axonal neuropathy [3-6]. Additionally, the disease severity and prolonged recovery can lead to stress reactions and consequent psychiatric symptoms [7,8]. Although elderly GBS patients are more likely to develop depression, anxiety, and other psychiatric symptoms during a prolonged clinical course, the specific psychiatric symptoms in elderly GBS patients remain unclear. Herein, we report three elderly patients with GBS who exhibited severe neurological deficits early in their clinical course, necessitating mechanical ventilation, and displayed akinesia in the chronic phase.

## Case presentation

Patient 1

Patient 1 was an 81-year-old man who presented with diplopia and unsteady standing three days after an upper respiratory infection. The next day, he was unable to move his eyes in any direction and presented dysarthria, bilateral ptosis, ataxia, and weakness of the upper extremities. Two days after onset, he showed flaccid tetraplegia with areflexia and was mechanically ventilated with intubation. A nerve conduction study (NCS) performed on day three after the onset of neurological symptoms revealed that the compound motor action potentials (CMAPs), distal latencies, and motor conduction velocities (MCVs) were within normal limits in all four limbs. However, F-wave responses showed reduced occurrence rates in the upper limbs. Sensory nerve action potentials (SNAPs) are absent in any of the four limbs. In subsequent nerve conduction studies, the CMAP amplitudes in all four limbs gradually decreased. However, there was no reduction in MCVs. The frequency of F-wave occurrence in the upper limbs increased, although with prolonged latencies. These findings were consistent with predominantly axonal neuropathy with demyelinating features. His cerebrospinal fluid (CSF) revealed albuminocytologic dissociation (CSF protein of 1,216 mg/dL with six white cells). In searching for a panel of anti-ganglioside antibodies (AGAs), anti-GT1a, GQ1b, GD1b IgG, and GM1 IgM antibodies were positive (Table 1).

**Table 1:** The panel of anti-ganglioside antibodies

Contents: Anti-GM1, GM2, GM3, GD1a, GD1b, GD3, GT1b, GQ1b, Gal-C, GalNAc-GD1a, and GT1a antibodies, including IgM, IgG
Patient 1: Positive: GT1a, GQ1b, GD1b IgG; GM1 IgM
Patient 2: All negative
Patient 3: Positive: GD1a, GD3, GM1 IgG; GalNAc-GD1a IgM Positive for IgG mixed with phosphatidic acid only: GD1b, GT1a, GT1b IgG

He was diagnosed with GBS and initially treated with plasmapheresis (PE), followed by intravenous immunoglobulin (IVIG).

**Figure 1:**
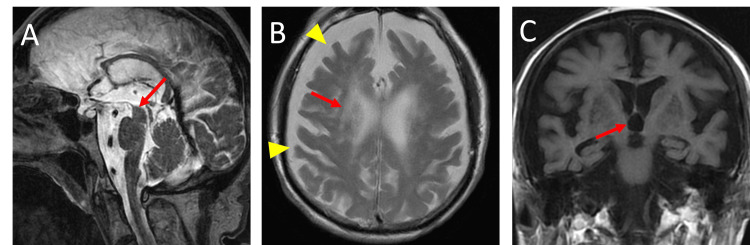
Brain magnetic resonance imaging of patient 1 (A) T2-weighted sagittal image shows midbrain atrophy (arrow). (B) T2-weighted horizontal image shows diffuse cortical atrophy (arrowheads) with an ischemic change in deep white matter (arrow). (C) T1-weighted coronal image shows the dilation of the third ventricle (arrow).

Four months after onset, the strength of extraocular and facial muscles slightly recovered, and he was transferred to our hospital. Later, his eye movement almost recovered. He was weaned off ventilation and could walk with a walker 11 months after onset. However, 27 months after onset, he gradually lost motivation. He refused walking training and showed limited eye movement, akinesia, limb and axial rigidity, and retrocollis that did not respond to L-dopa. Brain MRI showed mild midbrain atrophy (Hummingbird sign), and we diagnosed him with progressive supranuclear palsy (PSP) (Figure 1). At the age of 87, he died due to pneumonia. We did not obtain his autopsy.

Patient 2

**Figure 2:**
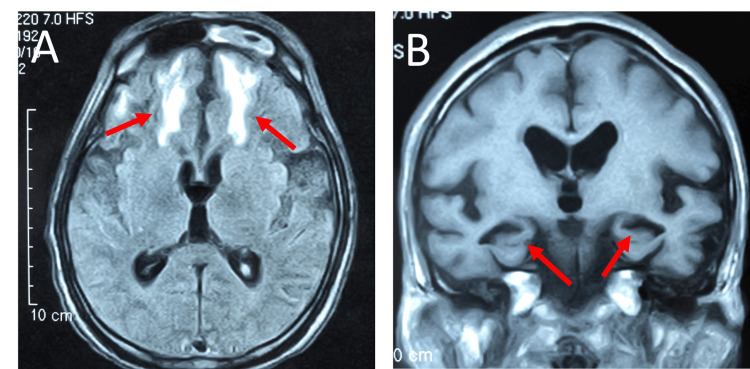
Brain magnetic resonance imaging of patient 2 (A) Fluid-attenuated inversion recovery (FLAIR) image shows hyperintensities in the periventricular white matter around the anterior horn of the lateral ventricles (arrows). (B) T1-weighted coronal image shows mesial temporal lobe atrophy (arrows).

Patient 2 was a 67-year-old man who presented with gait disturbance eight days after developing abdominal pain and diarrhea. The next day, the weakness of all four extremities rapidly progressed. Two days after onset, he suddenly suffered respiratory arrest and was mechanically ventilated with intubation. On neurological examination, both his eyes were fixed at the median. Light reflex was bilaterally dull, though pupil diameter was 5 mm. He did not show voluntary ocular and facial movements. He showed complete flaccid tetraplegia with areflexia. The NCS could not induce the CMAP and SNAP in routine nerves, including facial nerves. His CSF revealed albuminocytologic dissociation. No AGAs were detected when searching for a panel of AGAs (Table 1). He was diagnosed with GBS and initially treated with PE, followed by IVIG. He improved in extraocular, facial, and cervical movement and was transferred to our hospital eight months after onset. At that time, he could express his intentions through facial expressions and head movements. Subsequently, during physical therapy, he began to show signs of refusal with expressions of distress and shaking of the head. Around 11 months after onset, his voluntary movement gradually decreased, and he finally presented with akinetic mutism. Brain MRI revealed bilateral mesial temporal lobes atrophy and T2/ fluid-attenuated inversion recovery (FLAIR) hyperintensity in frontal white matter (Figure 2). At the age of 69, he died due to septic shock from gangrene in his foot. We did not obtain his autopsy.

Patient 3

Patient 3 was a 78-year-old man who presented with diplopia and tingling in both arms four days after enterocolitis-like symptoms. Two days later, his eyes were fixed at the median, and he showed flaccid tetraplegia. He was admitted to another hospital. He was intubated and mechanically ventilated. On day three, after the onset of neurological symptoms, the NCS of the right upper limb showed decreased CMAP and SNAP amplitudes, mildly prolonged distal latency in the median and ulnar nerves, and reduced motor and sensory conduction velocities in the median nerve (Table 2).

**Table 2:** The results of the nerve conduction study on day three after the onset in patient 3 Rt, right.

Nerve	Stimulation	Latency (ms) (reference values)	Amplitude (reference values)	Velocity (m/s) (reference values)
Motor			(mV)	
Rt. median	Wrist	4.7 (< 4.0)	1.8 (> 3.95)	
	Elbow		0.4	37.9 (> 54.3)
Rt. ulnar	Wrist	4.0 (< 3.1)	1.7 (> 4.22)	
	Elbow		0.6	57.1 (> 55.5)
Sensory			(μV)	
Rt. median	Wrist	3.3 (< 2.9)	2.1 (> 13.86)	
	Elbow		0.5	54.9 (> 58.3)
Rt. ulnar	Wrist	3.2 (< 2.4)	1.1 (> 10.77)	
	Elbow		0.6	60.5 (> 58.9)

By day 20, the NCS revealed the absence of the CMAP in the right median nerve, decreased CMAP amplitudes in the right ulnar and tibial nerves with reduced MCVs, and prolonged distal latencies. SNAPs were absent in all examined nerves (Table 3).

**Table 3:** The results of the nerve conduction study on day 20 after the onset in patient 3 NR, no response; Rt, right.

Nerve	Stimulation	Latency (ms) (reference values)	Amplitude (reference values)	Velocity (m/s) (reference values)
Motor			(mV)	
Rt. median	Wrist	NR		
	Elbow	NR		
Rt. ulnar	Wrist	5.3 (< 3.1)	0.1 (> 4.22)	
	Elbow		0.1	33.9 (> 55.5)
Rt. tibial	Ankle	7.5 (< 5.7)	2.4 (> 7.28)	
	Popliteal fossa		0.5	36.7 (> 43.9)
Sensory			(μV)	
Rt. median	Wrist	NR		
	Elbow	NR		
Rt. ulnar	Wrist	NR		
	Elbow	NR		
Rt. sural	Ankle	NR		

**Figure 3:**
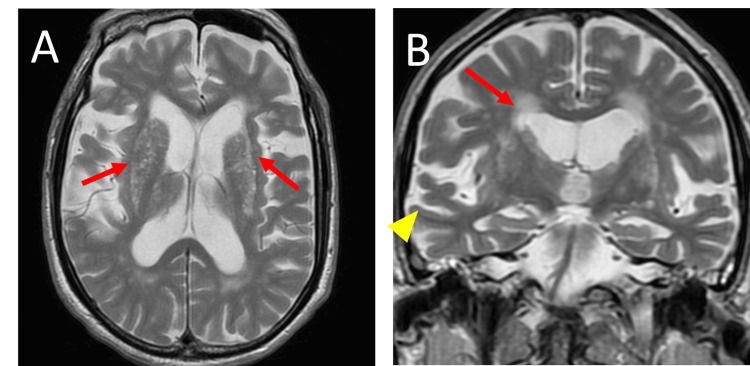
Brain magnetic resonance imaging of patient 3 (A) T2-weighted horizontal image and (B) coronal image show T2 hyperintensity (arrows) in periventricular white matter, basal ganglia, and temporal lobe atrophy (arrowhead).

These findings were consistent with predominantly axonal neuropathy with demyelinating features. CSF revealed albuminocytologic dissociation. In searching for a panel of AGAs, anti-GD1a, GD3, GM1 IgG, and GalNAc-GD1a IgM antibodies were positive (Table 1). Anti-GD1b, GT1a, and GT1b IgG antibodies were positive only with phosphatidic acid (Table 1). He was diagnosed with GBS and initially treated with PE, followed by IVIG. Two months after onset, he was transferred to our hospital and weaned off ventilation immediately. He could move his eyes without limitation, speak via a tracheostomy tube, and lift his arms and legs slightly. However, six months after onset, he quickly became angry and refused physical therapy and medical care. Later, he presented with limited vertical movement of both eyes, akinesia, and limb contracture. Brain MRI revealed bilateral temporal lobes atrophy and T2 hyperintensity in periventricular white matter and basal ganglia (Figure 3). At the age of 80, he died due to renal failure. We did not obtain his autopsy.

## Discussion

We found two crucial clinical points. First, we highlighted the clinical features in elderly patients with GBS. Epidemiological research from Western countries shows a 20% increase in GBS incidence with each decade of age, with a higher predisposition in males compared to females [2]. Similarly, research in a local area in Japan shows a rise in incidence among older adults, suggesting this trend is geographically widespread [3]. The clinical features in elderly patients with GBS demonstrated in previous reports were consistent with our cases. The first feature is that elderly patients have a shorter time from onset to peak severity, greater disease severity, and a longer time to recovery, resulting in more extended hospital stays [3-6]. This feature was also true for our patients, who exhibited severe neurological deficits early in their clinical course, necessitating mechanical ventilation. In addition, elderly GBS patients are more prone to cranial nerve involvement, such as facial palsy and bulbar symptoms. Our patients also showed cranial nerve involvement, but the development of ocular movement deficits was unique. Although antecedent illness is a hallmark suggesting GBS, elderly patients have fewer antecedent illnesses and a longer interval between the antecedent infection and the onset of symptoms [5,6]. In contrast, all our patients had antecedent illnesses and a short interval of 2-8 days between the onset of symptoms, indicating variability in the prodromal phase among elderly individuals.

The second feature in elderly patients with GBS is the laboratory findings. Elderly patients tend to have a more pronounced immunoglobulin abnormality in the CSF; albuminocytologic dissociation in all our patients highlights this aspect [3,5]. The presence of axonal neuropathy in NCSs, more prevalent in elderly patients, was also evident in our patients [3,4,6]. The predominance of axonal neuropathy may be associated with slower recovery in elderly patients [3,4,6]. The role of AGAs in elderly patients remains unclear, as no reports show an association between aging and the frequency or specificity of these antibodies. Among the positive AGAs found in patient 1 and patient 3, we identified anti-GM1 and GD1a antibodies, which are associated with acute motor axonal neuropathy (AMAN) and acute motor-sensory axonal neuropathy (AMSAN) [9]. Kaida et al. implicate an anti-GD1b antibody in sensory ataxic neuropathy, while reports link anti-GalNAc-GD1a IgG antibody to facial palsy, especially following Cytomegalovirus infection [10,11]. An anti-GD3 antibody is linked to acute bulbar palsy plus syndrome, a variant of GBS [12]. Anti-GQ1b antibody is frequently detected in Miller-Fisher syndrome and is strongly associated with external ophthalmoplegia and ataxia. An anti-GT1a antibody relates to the pharyngeal-cervical-brachial variant of GBS. These antibodies may have influenced the clinical courses of patient 1 and patient 3, contributing to the predominantly axonal neuropathy with demyelinating features and cranial nerve involvement seen in both cases. In particular, we could not rule out the possibility of Miller-Fisher syndrome overlap in patient 1, who showed external ophthalmoplegia and a positive anti-GQ1b antibody.

The second crucial clinical point is that all our patients displayed akinesia in the chronic phase of GBS. MRI in Patient 1 showed midbrain atrophy (Hummingbird sign), leading to a diagnosis of PSP, though we did not confirm it with autopsy. In contrast, MRI findings in patient 2 and patient 3 revealed temporal lobe atrophy, suggesting underlying neurodegenerative changes. Additionally, all patients had T2 hyperintensities in the cerebral white matter and basal ganglia, likely reflecting ischemic lesions due to cerebrovascular disease. These abnormalities were likely unrelated to GBS but may have contributed to the patients' diminished psychomotor activity. Most GBS patients, except for those with Bickerstaff brainstem encephalitis, a GBS variant, show normal MRI findings [13]. Therefore, given the increased susceptibility of elderly patients to both neurodegenerative and cerebrovascular disorders, it is plausible that our patients had pre-existing cerebrovascular or neurodegenerative conditions, which complicated their clinical course [14].

Furthermore, Tzeng et al. report that adult patients with GBS have a 4.3-fold increased risk of developing psychiatric disorders in the long duration of follow-up [7]. Our patients' refusal of physical therapy during the recovery phase may also have been a manifestation of their psychiatric symptoms. Psychiatric assessments often reveal significant symptoms in patients with GBS, including sadness, anxiety, and a notable reduction in psychomotor speed and facial expressivity [7,8,15]. Traumatic stress from acute life-threatening illness and long-term disabilities in the chronic (recovery) phase may contribute to the development of psychiatric symptoms in patients with GBS [7,16,17]. Therefore, neurologists must consider the potential for coexisting psychiatric and neurological symptoms in elderly patients with GBS, which may necessitate integrated psychiatric management. In addition to clinical observation, standardized screening tools such as the Mini-Mental State Examination (MMSE), Geriatric Depression Scale (GDS), and Hospital Anxiety and Depression Scale (HADS) are essential for early identification of psychiatric symptoms. Neuroimaging techniques such as head computed tomography, brain MRI, and cerebral blood flow single-photon emission computed tomography can also detect underlying structural or functional brain changes that may contribute to psychiatric and cognitive disturbances. Timely intervention, including the use of selective serotonin reuptake inhibitors and psychotherapy, can help alleviate these challenges and improve the overall prognosis in elderly GBS patients [15].

## Conclusions

We highlighted the clinical features in elderly patients with GBS. Our patients shared many clinical features with elderly GBS patients, as demonstrated in previous reports. However, our patients were unique in having a short interval between antecedent illness and the onset of symptoms, as well as ocular movement deficits. Furthermore, our patients displayed refusal of physical therapy in the chronic phase and eventually showed akinesia. Long-term management strategies, both neurological and psychiatric, are essential for elderly GBS patients.
